# Therapeutic Potential of Terpenoids in Cancer Treatment: Targeting Mitochondrial Pathways

**DOI:** 10.1002/cnr2.70006

**Published:** 2024-09-05

**Authors:** Jianxin Guo, Ming Huang, Shuang Hou, Jianfeng Yuan, Xiaoyue Chang, Shuang Gao, Zhenhan Zhang, Zhongbing Wu, Jing Li

**Affiliations:** ^1^ College of Integrated Chinese and Western Medicine Hebei Medical University Shijiazhuang China; ^2^ The Fourth Hospital of Hebei Medical University Shijiazhuang China

**Keywords:** cancer, cell death, mitochondrial, terpenoids

## Abstract

**Background:**

In recent decades, natural compounds have been considered a significant source of new antitumor medicines due to their unique advantages. Several in vitro and in vivo studies have focused on the effect of terpenoids on apoptosis mediated by mitochondria in malignant cells.

**Recent findings:**

In this review article, we focused on six extensively studied terpenoids, including sesquiterpenes (dihydroartemisinin and parthenolide), diterpenes (oridonin and triptolide), and triterpenes (betulinic acid and oleanolic acid), and their efficacy in targeting mitochondria to induce cell death. Terpenoid‐induced mitochondria‐related cell death includes apoptosis, pyroptosis, necroptosis, ferroptosis, autophagy, and necrosis caused by mitochondrial permeability transition. Apoptosis and autophagy interact in meaningful ways. In addition, in view of several disadvantages of terpenoids, such as low stability and bioavailability, advances in research on combination chemotherapy and chemical modification were surveyed.

**Conclusion:**

This article deepens our understanding of the association between terpenoids and mitochondrial cell death, presenting a hypothetical basis for the use of terpenoids in anticancer management.

**Abbreviations**
Akt: AKT serine/threonine kinase
ATO: arsenic trioxide
ATP: adenosine triphosphate
BA: betulinic acid
BAD: BCL2 associated agonist of cell death
Bax: BCL2‐associated X
BCL‐2: B‐cell lymphoma‐2
CAV‐1: caveolin‐1
CDDP: chemotherapeutic agent cisplatin
DHA: dihydroartemisinin
ERK: extracellular regulated protein kinases
ERS: endoplasmic reticulum stress
GPX4: glutathione peroxidase 4
GSH: glutathione
HA: hyaluronic acid
HK‐II: hexokinase II
IAPs: inhibitors of apoptosis proteins
IKKβ: Inhibitor of kappa B kinase
JNK: c‐Jun *N*‐terminal kinase
JNK: c‐Jun *N*‐terminal kinase
MAPK: mitogen‐activated protein kinase
Mcl‐1: myeloid cell leukemia‐1
MLKL: mixed lineage kinase domain‐like protein
MMP: mitochondrial membrane potential
mtor: mammalian target of rapamycin
Myc: MYC proto‐oncogene, BHLH transcription factor
NDDS: nanoscale drug delivery systems
NDUFS3: NADH: Ubiquinone Oxidoreductase Core Subunit S3
NF‐kB: nuclear factor‐kappa B
NRF2: nuclearrespiratoty factor 2
OA: oleanolic acid
Ori: oridonin
OSCC: oral squamous cell carcinoma
OXPHOS: oxidative phosphorylation
P38: P38 mitogen‐activated protein kinase
PARP: poly ADP‐ribose polymerase
PDAC: pancreatic ductal carcinoma
PI3K: phosphatidylinositol 3‐hydroxy kinase
PINK1: PTEN induced putative kinase 1
PTL: parthenolide
Ras: rat sarcoma
RIP1: receptor interacting protein 1
RIPK1: receptor interacting serine/threonine kinase 1
RIPK3: receptor interacting serine/threonine kinase 3
RNA: ribonucleic acid
ROS: reactive oxygen species
SDHB: succinate dehydrogenase complex iron sulfur subunit B
SIRT‐1: silent information regulator‐1
SLC7A11: solute carrier family 7 member 11
STAT: signal transducer and activator of transcription
TPL: triptolide
TPS: terpene synthase
TrxR: thioredoxin reductase
UQCRFS1: ubiquinol‐cytochrome c reductase, rieske iron–sulfur polypeptide 1

## 1 Introduction

Cancer, the leading cause of death worldwide, has long been a major public health concern. The American Cancer Society estimates 2 001 140 new cancer cases and 611 720 cancer deaths to occur in the United States by 2024 [1]. With a growing and aging population, cancer treatment is currently more challenging than ever [2, 3, 4].

Unlike well‐regulated healthy cells, tumor cells require a significant amount of energy to sustain their rapid growth [5, 6]. Mitochondria, a junction in the metabolic pathways of glucose, glutamine, and lipids, and the powerhouse in eukaryotic cells can meet the energy demand of tumor cells [7, 8]. Paradoxically, mitochondria are a key organelle in programmed cell death [9], regulating cell death through various mechanisms, such as ferroptosis, mitophagy, NETosis, entosis, pyroptosis, necroptosis, parthanatos, apoptosis, clockophagy, alkaliptosis, autosis, and oxeiptosis [10, 11, 12, 13]. These mitosome‐dependent cell death pathways can be programmed to treat cancer [14, 15, 16].

**FIGURE 1:**
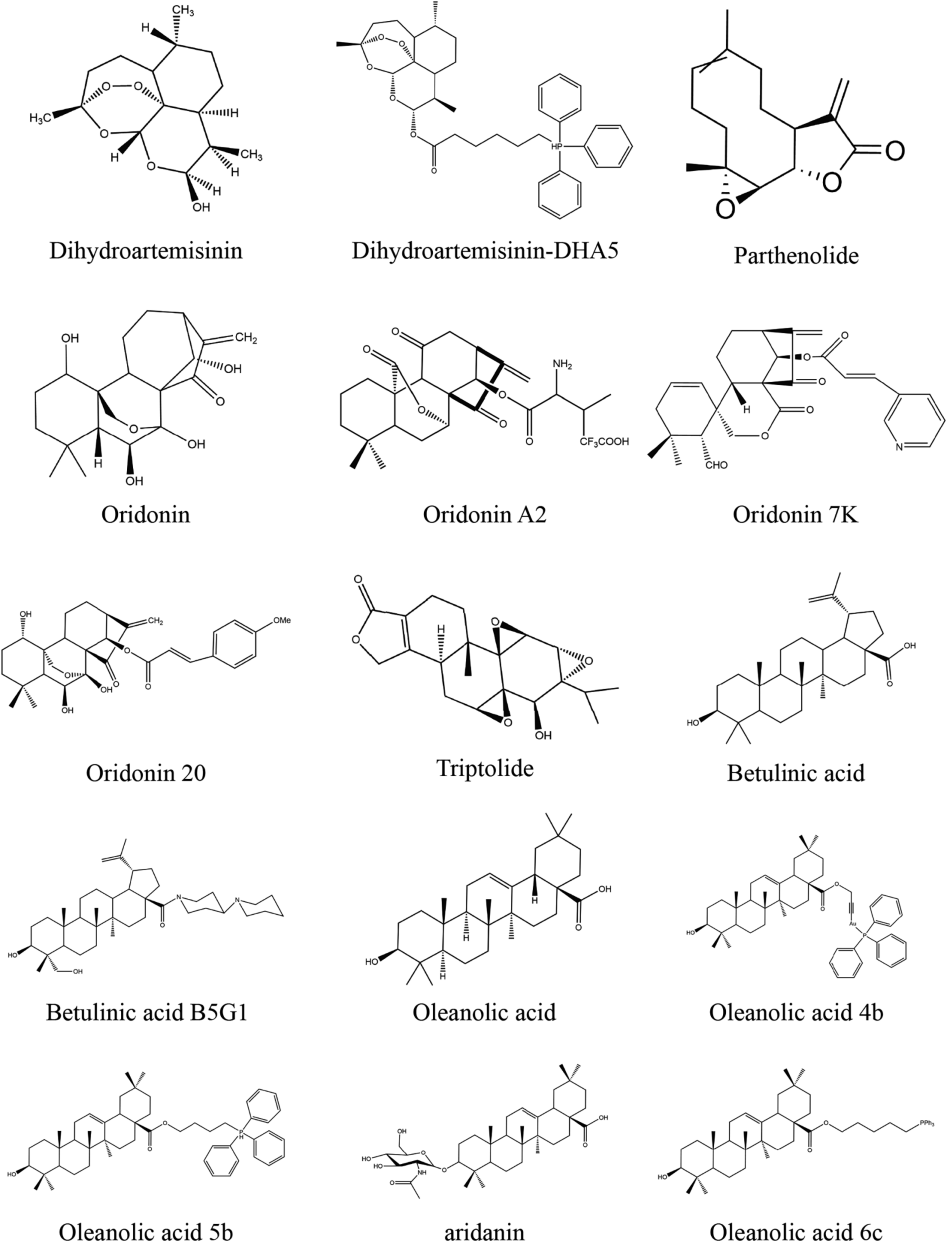
Chemical structures of selected terpenoids and their derivatives.

Natural compounds provide unique advantages in cancer treatment due to their structural diversity and multifunctional activities [17, 18, 19, 20]. Several studies have revealed that terpenoids are effective in treating cancer through the mitochondria‐dependent cell death pathways [21, 22, 23, 24, 25]. Terpenoids are the most extensive category of naturally occurring chemical substances, including over 40 000 chemicals. They are frequently used in the taste, perfume, chemical, and medicinal sectors [26, 27, 28]. Terpenoids may be categorized into several groups based on their structure, including tetraterpenoids, diterpenoids, monoterpenoids, hemiterpenoids, sesquiterpenoids, sesterterpenoids, triterpenoids, and polyterpenoids [29]. In recent years, terpenoids have been widely investigated for their potential applications in human pathophysiology due to their diverse biological activities [30, 31]. An increasing number of pharmacological investigations have reported the advantages of terpenoids in tumor therapy [32], and further studies of these compounds may help develop antitumor drugs. In this review article, we focused on natural terpenoids that exert anticancer activity through the mitochondrial cell death pathway. In addition, we selected three terpenoids that our extensive literature search revealed to be the most studied in cancer‐related mitochondrial cell death. In particular, we investigated two diterpenoids (oridonin [Ori] and triptolide [TPL]), two sesquiterpenoids (dihydroartemisinin [DHA] and parthenolide [PTL]), and two triterpenoids (betulinic acid [BA] and oleanolic acid [OA]) as well as some of their derivatives (Figure 1).

## 2 Diversity of Terpenoids and Their Sources

Terpenoids are an extensive library of compounds for anticancer drug screening [33]. In general, terpenoids are derived from isoprene units. The condensation of two isoprenyl units results in monoterpene, three units form a sesquiterpene, four units form a diterpene, and six units form a triterpene; the isoprenyl units involved in the condensation process are usually in the form of diphosphates [34]. Terpenoids are diverse for several reasons. First, variation in the terpene skeleton, whose synthesis is catalyzed by terpene synthase (TPS), can lead to changes in terpenoids [35, 36]. Second, individual amino acid changes in TPS can cause drastic alterations in the resulting terpenoid structure [37]. Moreover, mutation in TPS genes is facilitated by their existence in families of 30–100 genes [38].

Studies have revealed that terpenoids are ancient compounds that had a part in the early lifeform evolution [39]. Various range of organisms, such as terrestrial plants and animals [40, 41], microorganisms [42, 43], marine plants and animals [44, 45], and even some organisms living in the harsh environment of the Arctic and Antarctica, synthesize terpenoids [46]. However, our demand for the more potent varieties remains to be met despite the large supply of naturally generated terpenoids [47, 48]. Industrial production of terpenoids can take one of three possible routes. The most direct approach is simply extraction from natural resources, including plants and animals [49, 50, 51]. Another possibility is chemical synthesis [52, 53]. However, the former is no longer considered a viable option due to environmental concerns. In addition, chemical synthesis is infeasible due to the structural complexity of the products [54]. The third approach—microbial cell factory—now holds more promise due to rapid advances in synthetic biology and metabolic engineering. These include engineered cyanobacteria [55], engineered phototrophic bacteria [56], engineered yeast [57], and *Yarrowia lipolytica* cell factories [58]. Microbial cell factories, known for their robustness, safety, and sufficient supply of precursors, achieved de novo synthesis of terpenoids. This offers a promising and environmentally friendly alternative to natural plant extraction and traditional terpenoid supply methods, such as chemical synthesis. This advancement paves the way for the use of terpenoids as antineoplastic drugs.

## 3 Sesquiterpenoids in Mitochondrial Cell Death

### 3.1 DHA in Mitochondrial Cell Death

DHA is the main active compound in the sesquiterpenoid artemisinin. It was initially used in treating malaria upon its discovery. DHA exerts its antimalarial effects by damaging the membranes of the digestive vacuoles and mitochondria of the parasite [59]. Investigations on the metabolism of drugs have demonstrated DHA as the primary active form of many artemisinin derivatives, including artesunate, artemether, and arteether. This indicates that DHA is an active component responsible for the effects of these medications [60, 61, 62]. In addition to antimalarial effect, DHA has demonstrated significant antitumor activity both in vitro and in vivo [63, 64], which is believed to involve mitochondria‐related cell death.

DHA has impeded cell growth and prompted ferroptosis in lung malignancy and leukemia cells by disrupting mitochondrial functions. Specifically, DHA has been revealed to disturb the membrane potential of the mitochondria, increase cytoplasmic levels of reactive oxygen species (ROS), and decrease the key protein expression related to Fe‐S cluster binding within mitochondrial complexes, namely NDUFS3, SDHB, and UQCRFS1, as well as SLC7A11, which is an iron‐dependent cell death pivotal modulator [65, 66]. The synergistic application of DHA and the chemotherapeutic agent cisplatin (CDDP) disrupts mitochondrial homeostasis in pancreatic ductal carcinoma cells. This disruption is evidenced by mitochondrial morphology changes, respiratory capacity reduction, ATP production decrease, and mitochondrial ROS accumulation. These effects cause the free iron and lipid peroxidation buildup, ultimately causing ferroptosis [67]. Interestingly, DHA administration in combination with CDDP induced significant mitochondrial autophagy in oral squamous cell carcinoma (OSCC) [68]. Meanwhile, DHA alone caused mitochondrial autophagy in liver carcinoma cells (HepG2215) and gastric cancer cells (SGC7901/DDP) [69, 70]. Notably, autophagy has a dual role of cytoprotection and cytotoxicity, the specific role of which depends on the kind of tumor cell and stress level [71]. Wu et al. revealed that applying DHA to lung cancer cells (A549) reduced radiation‐induced mitochondrial autophagy and radioresistance [72]. The tumor‐suppressing effects of DHA are more prominently observed in mediating mitochondrial apoptosis in addition to inducing ferroptosis and mitochondrial autophagy. Investigations have revealed that DHA causes mitochondrial apoptosis in various human malignancy cell lines, including ovarian cancer (IOSE80, SKOV3, A2780, OVCAR3, and TOV112D), melanoma (B16F10), breast cancer (4T1), lung cancer (A549), bladder cancer (T24), and neuroblastoma (SH‐SY5Y) [73, 74, 75, 76, 77, 78]. Interestingly, a clinical study revealed that apoptosis was present in 67% of the tumor cells of patients with colorectal cancer in the artesunate group. Additionally, Ki67 expression decreased and CD31 expression increased in patients in the artesunate group [79].

Scientists have developed various nanoscale drug delivery systems to accommodate the poor stability of DHA, reduced solubility of water, and short plasma half‐life [80]. These include polymer‐based nanoplatforms for DHA–hyaluronic acid conjugates [81], lipid‐based nanoplatforms for R8 modified epirubicin‐DHA liposomes [82], metal–organic framework‐based nanoplatforms for loading DHA [83], and inorganic nanoparticle‐based platforms for delivering DHA and transferrin [84].

### 3.2 PTL in Mitochondrial Cell Death

PTL is a sesquiterpene lactone found in the branches of the plant *Tanacetum parthenium*, and it demonstrates potent anticancer and anti‐inflammatory activities [85]. Intrigued by its undeniable anticancer effect, many scientists have been trying to unravel the underlying mechanism.

Investigations have revealed that PTL induces cancer cell death primarily by affecting mitochondria [86]. In hepatocellular carcinoma stem cells, PTL induces ROS production, decreases mitochondrial membrane potential (MMP) and oxidative phosphorylation (OXPHOS) levels, stimulates cytosolic stop in the G1 phase, and induces apoptosis [87]. Interestingly, in hepatocellular carcinoma cells (HEK293T, HepG2, MHCC 97H, and Huh7), PTL administered in combination with arsenic trioxide (ATO) improved cytoprotective autophagy via the PI3K/Akt/mTOR pathway as well as induced mitochondrial apoptosis through the classical pathway [88]. This observation hints at a complex association between apoptosis and autophagy, with the latter analogous to a double‐edged sword whose inhibition can potentially improve the therapeutic effect of PTL and ATO [89]. Autophagy has demonstrated the ability to suppress apoptosis. Reports have indicated mitophagy as the main mechanism underlying this phenomenon, and after mitochondrial damage, proapoptotic factors increase the permeability of the membranes of the affected mitochondria, thereby activating autophagy, which eliminates damaged mitochondria and reduces the chance of apoptosis [90]. In addition, autophagy activates apoptosis in certain special cases. In particular, the formation of autophagic vesicles may potentially trigger caspase activation. Moreover, autophagy breaks down inhibitors of apoptosis proteins (IAPs) [91, 92]. PTL in cells of hepatocellular carcinoma modifies their antioxidant microenvironment via thiol changes, making the tumor cells more sensitive to increased ROS levels, thereby causing lipid peroxidation and eventually ferroptosis [93].

Another investigation revealed that PTL has triggered apoptosis in all lymphatic tumor cell lines, including KOPN‐8, Farage, Raji, 697, NCI‐H929, CEM, and MOLT‐4. Apoptosis was accompanied by a significant increase in ROS as well as moderately low glutathione and MMP levels. Furthermore, PTL effectively suppressed the stimulation of nuclear factor kappa B (NF‐kB) in CEM and MOLT‐4 cell lines [94]. An in vitro and an in vivo model of triple‐negative breast malignancy confirmed similar outcomes [95]. NF‐κB affects mitochondrial respiration and mitochondrial dynamics, whereas mitochondria respond by promoting NF‐κB activation. The interaction between the two sustains tumor cell survival and development [96]. Additionally, PTL targets multiple key proteins that act on the NF‐κB signaling pathway, such as receptor‐interacting protein 1 (RIP1) [95, 97], NF‐κB inhibitor (IκB), and kappa B kinase inhibitor (IKKβ) [98]. This provides a new perspective on the clinical application of PTL in tumor treatment.

## 4 Diterpenoids

### 4.1 Oridonin in Mitochondrial Cell Death

Ori is a biologically active diterpenoid extracted from *Isodon rubescens* (Hemsl.) H. Hara with potent antitumor properties [99]. Ori induces apoptosis and necroptosis in acute myeloid leukemia cells [100]. Reports indicated that apoptosis caused by Ori, which depends on mitochondria, is associated with AKT suppression via phosphorylation [101, 102, 103]. AKT, a serine/threonine kinase, has a vital role in several essential physiological mechanisms, such as survival, growth, invasion, and apoptosis. It has exhibited potential as a target for cancer treatment [104]. Ferroptosis has been identified as another mode of Ori‐induced malignancy cell death. A2, a derivative of Ori, decreased MMP, lowered BCL‐2 levels, promoted the PARP and Caspase‐3/9 proteins cleavage, and induced mitochondria apoptosis in vitro and in vivo models of gastric malignancy. This increased intracellular ROS, reduced GPX4, catalyzed lipid peroxides reduction, and eventually caused ferroptosis [105].

To enhance the antitumor impact of Ori and explore its pathway of action, synthetic derivatives have become a common tool [106]. Ni et al. created and produced a range of Ori spirolactone‐type and enmein‐type compounds (Ori 7K) with various C‐14 hydroxyl substitution levels [107]. Another study investigated a series of Ori derivatives, such as acetylated derivatives (Ori 20) [108]. Interestingly, both studies revealed that Ori derivatives caused apoptosis in tumor cells by increasing intracellular ROS levels and depolarizing MMP. The IC_50_ values of these derivatives revealed a substantial decrease compared with Ori.

Furthermore, Ori has exhibited significant efficacy when used in combination with conventional antitumor medicines in clinical settings. In particular, the combined application of Ori with homoharringtonine downregulated c‐KIT and its associated downstream signaling pathways (STAT, MAPK, and PI3K), thereby reducing MMP, decreased Mcl‐1 levels, and Caspase‐3 activation, ultimately causing cellular apoptosis [109]. This emphasizes the significance of using combination therapies for cancer management. The synergistic effect of Ori in combination with chemotherapy and targeted therapy has improved the cancer cells' drug sensitivity, elevated cellular mitochondrial apoptosis, reduced drug dosage, and mitigated side effects [110, 111]. Furthermore, the literature indicates that Ori's anticancer properties may be related to its regulation of mitochondrial pyroptosis and have been used as a new targeted therapeutic approach [112].

### 4.2 TPL in Mitochondrial Cell Death

TPL, a diterpenoid [113], is the predominant pharmacologically active component of *Tripterygium wilfordii* Hook. F. It is used in treating inflammatory [114, 115, 116], autoimmune [117, 118], renal [119, 120], and neurological diseases [121, 122] as well as tumors [123, 124].

Compelling evidence from studies using ex vivo and in vivo tumor models has indicated that TPL induces apoptosis in colorectal, non‐small cell lung, and pancreatic cancers and acute myeloid leukemia cells via the pathway of mitochondrial apoptosis [125, 126, 127, 128]. Both in vitro and in vivo models of malignant cells indicated that TPL significantly inhibited the NF‐κB signaling pathway, SIRT‐1/CAV‐1 axis, and phosphorylation of RNA pol II; elevated mitochondrial fission and ROS; reduced MMP; and induced changes in apoptosis‐related proteins, such as Caspase‐9, Caspase‐3, Bcl‐2, Bax, C‐PARP, c‐Myc, and Mcl1. In head and neck tumor cells, TPL triggers apoptosis of the mitochondria by inhibiting mitochondrial hexokinase II (HK‐II), a critical metabolic enzyme in the glycolytic pathway, thereby activating the apoptosis‐associated BAD/BAX‐Caspase 3 pathway [129]. In addition, TPL‐induced suppression of NRF2 expression causes ferroptosis in leukemia cells [130]. Interestingly, in in vitro head and neck tumor cells, ferroptosis was considered to be caused by TPL in synergy with erastin, which inhibited the NRF2/SLC7A11 axis [129]. NRF2 plays a crucial role in reducing lipid peroxidation and iron‐induced cell death, indicating that targeting NRF2 with TPL may have clinical implications [131]. In addition, a Phase I study involving patients with advanced gastrointestinal tract cancer initially assessed the pharmacokinetics and antitumor activity of Minnelide, a water‐soluble prodrug of TPL [132]. This study revealed that Minnelide was converted to TPL and reached its peak concentration 30 min after infusion, with a half‐life of ~1 h, a reduction in mean target lesion tumor density in 16 out of 28 (57.1%) patients, and a disease control rate lasting from 2 to 6 months in 14 out of 28 (50%) evaluable patients.

However, the medical efficacy of TPL is constrained by its organ toxicity, particularly hepatotoxicity [133]. Research indicates that the hepatotoxic effects of TPL may be associated with mitochondrial oxidative stress [134], excessive mitochondrial autophagy [135, 136], and mitochondrial apoptosis [137]. Recent studies have revealed that mitochondria‐targeting derivatives of TPL can mitigate their renal and hepatic toxicity [138], thereby bolstering the argument for clinical applications of TPL as an antitumor drug. However, additional investigation is warranted to confirm the dual effects and underlying pathways of TPL on mitochondrial death in tumor cells.

## 5 Triterpenoids

### 5.1 BA in Mitochondrial Cell Death

BA, a pentacyclic triterpenoid primarily detected in birch, sycamore, and other plants [139, 140], has revealed a range of biological features such as antitumor [141], anti‐inflammatory [142], neuroprotective [143], and antioxidant effects [144]. Recently, several studies have revealed that the anticancer mechanism of BA correlates with its cellular mitochondrial apoptosis induction, which applies to a range of human cancer cell lines, including laryngeal cancer cells originating from the head and neck region (AMC‐HN‐8), gastrointestinal malignancy cholangiocarcinoma cells (NOZ), colorectal cancer cells (HCT116), ovarian cancer cells (A2780, SW480, and DLD‐1), and bladder cancer cells (T24, UMUC‐3, and 5637) [145, 146, 147, 148, 149]. These research results indicate that BA‐induced apoptosis is initiated through the intrinsic pathways mediated by mitochondria, as manifested as Caspase‐3/8/9 activation, PARP cleavage, Bax accumulation in mitochondria, MMP disruption, and ROS level elevation [147, 149]. However, Kim et al. revealed that BA‐mediated mitochondrial apoptosis in human bladder cancer cells had no effect on intracellular ROS levels [149], and this result was corroborated by Sharma and Kumar [150]. However, the mechanism of this ROS‐independent pathway of apoptosis warrants further investigation. Yao et al. [151] revealed that B5G1, a BA derivative, triggered apoptosis in malignancy cells that are resistant to many drugs, HepG2/ADM and MCF‐7/ADR, while activating the non‐canonical mitophagy pathway, PINK1/Parkin. This pathway facilitated mitochondrial autophagy in the cells, acting as a protective mechanism against apoptosis. This indicates that PINK1/Parkin‐mediated mitochondrial autophagy suppression increases drug sensibility in malignancy cells that are resistant to many drugs.

### 5.2 OA in Mitochondrial Cell Death

OA, a pentacyclic triterpenoid, chemically known as 3β‐hydroxy‐olean‐12‐en‐28‐oic acid, is detected both in its free form and as the glycosidic ligand of triterpenoid saponins. It has been identified in over 1620 species of edible and medicinal plants [152, 153, 154]. OA demonstrates various physiological properties, such as anti‐inflammatory [155, 156], hypoglycemic [157, 158], and anticholestatic effects [159]; moreover, it offers protection against renal damage [160]. Furthermore, OA exhibits notable anticancer properties in various tumor types through mitochondrial autophagy, apoptosis, and ferroptosis [161, 162, 163, 164]. Both in vivo and in vitro experiments have revealed that OA triggers autophagy and apoptosis mediated by mitochondria in hepatocellular carcinoma. This action may be associated with the suppression of NF‐κB, Akt, and mTOR pathways [165, 166].

Recent research has revealed that OA may cause mitochondrial apoptosis in tumor cells by activating the P38 pathway. OA suppresses the function of JNK [162] and modulates P38 activity in this apoptotic signaling cascade [167], thereby regulating downstream targets FOXO3a/Sirt6 [164]. This ultimately disrupts MMP, reduces ROS, releases cytochrome c, upregulates caspases‐3/7/8/9 and Bak, and downregulates Bcl‐2 levels [161, 162]. Furthermore, the OA derivative (OA 4b) suppresses thioredoxin reductase (TrxR) activity and induces endoplasmic reticulum stress through ROS activation, ultimately causing apoptosis in cancerous cells [168].

Studies have revealed that mitochondria are crucial in initiating apoptosis and are recognized as both a source and target of ROS [169]. OA partakes in multiple mitochondria‐mediated pathways, some involving ROS [170, 171] and others independent of ROS, in promoting tumor cell apoptosis [161]. This indicates that the antitumor effect of OA is not entirely dependent on the oxidation state, which aligns with the “double‐edged sword” characteristic of ROS.

Mitochondria are potential targets for cancer therapy. Several studies have developed and synthesized OA derivatives (5b, 6c, and aridanin) that specifically target mitochondria. Such mitochondria‐targeting derivatives selectively destroy malignancy cells by inducing apoptosis, ferroptosis, autophagy, and necrotic cell death [163, 172, 173] through a mechanism primarily involving the PI3K‐Akt pathway [174] (Tables 1 and 2).

**TABLE 1:** In vitro studies of terpenoids as inducers of mitochondrial death in tumor cells are shown in the table.

Terpenoids	Plant sources	Cancer model	Mechanism and effects	Reference
DHA	*Artemisia annua*	Human lung cancer cell lines including NCI‐H23 and XWLC‐05	↑ROS, ↑MDA, ↓PRIM2 /SLC7A11 Axis →Ferroptosis	[65]
DHA	*Artemisia annua*	Human acute promyelocytic leukemia cell line HL60, acute myeloid leukemia cell line KG1, and acute monocytic leukemia cell line THP‐1	↓MMP, ↑ROS, ↓NDUFS3, ↓SDHB, ↓UQCRFS1, ↓TFAM, ↓ClpP, ↑p‐AMPK, ↓mTOR/p70S6k signaling pathway →Ferroptosis	[66]
DHA + CDDP	*Artemisia annua*	Human oral normal and cancer cell lines, including normal oral keratinocyte (NOK), SAS, and HSC3.	↓Bcl‐2, ↑Bax, ↑Caspase‐3, ↑ROS ↑Leakage of Cyt‐C and AIF from mitochondria →Mitophagy	[68]
DHA	*Artemisia annua*	Human liver cancer cells HepG2215	↑ROS, ↑AIM2/Caspase‐1 inflammasome →Autophagy	[69]
DHA	*Artemisia annua*	Cisplatin (DDP)‐resistant gastric cancer cell strain SGC7901/DDP	↑Caspase‐3/8/9, ↓PI3K/AKT/mTOR, ↓p‐gp, ↑Beclin1, ↑LC3‐II →Mitophagy & Reversed the cisplatin resistance	[70]
DHA	*Artemisia annua*	Human lung cancer cell line A549	↓CIRBP, ↓PINK1/Parkin →Reduced Mitophagy and radioresistance	[72]
DHA	*Artemisia annua*	Human ovarian epithelial cells IOSE80 and several ovarian cancer cells, SKOV3, A2780, OVCAR3 and TOV112D	↓ROS, ↓p‐ERK, ↓p‐AKT, ↓ROMO1 →Apoptosis	[73]
DHA	*Artemisia annua*	Human melanoma cell line B16F10	↓Bcl‐2, ↑Bax, ↓p‐STAT3 →Apoptosis	[74]
DHA‐cinnamic acid hybrids	*Artemisia annua*	The human breast cancer cell line MCF‐7 and MDA‐MB‐231, human non‐small‐cell lung cancer cell line A549	↓PI3K/Akt/Bad, ↑Bax, ↓Bcl‐2, ↑Caspase‐3, ↑ROS, ↓MMP →Apoptosis	[76]
Mito‐DHA5	*Artemisia annua*	Human bladder cancer cell line T24	↓Bcl‐2, ↑Bax, ↓Cyt‐C, ↑Caspase‐3, ↓C‐PARP, ↑ROS, ↓MMP →Apoptosis	[77]
DHA	*Artemisia annua*	Human neuroblastoma cell line SH‐SY5Y	↑PARP‐1, ↑Caspase‐3, ↑γH2AX, ↑ROS, ↓MMP, ↑DSB →Apoptosis	[78]
PTL + ATO	*Tanacetum parthenium*	Hepatocellular carcinoma cell lines HEK293T, HepG2, MHCC 97H and Huh7	↓USP7‐HUWE1‐p53 axis, ↓PI3K/Akt/mTOR pathway↓Bcl‐2, ↑Bax, ↑ROS, ↓MMP →Protective autophagy and apoptosis	[88]
PTL	*Tanacetum parthenium*	Human breast cancer cell line MDA‐MB231	↑p‐ERK1/2, ↑p‐JNK, ↑RIP‐1, ↓NF‐κB, ↑LC3‐II, ↑Beclin‐1, ↑ROS, ↓MMP, ↑Ca2+ →Autophagy	[95]
Ori	*Rabdosia rubescens*	Human acute myeloid leukemia cell line OCI‐AML3, Human promyelocytic leukaemia cell line HL60, Human red leucocyte leukaemia cell line HEL	↑RIPK1‐Caspase‐8‐Caspase‐3 pathway, ↑RIPK1‐RIPK3‐MLKL pathway →Apoptosis and necroptosis	[100]
Ori	*Rabdosia rubescens*	Human esophageal squamous cancer KYSE‐30, KYSE‐150, EC9706 cell lines, and human esophageal epithelial cells (HEEC)	↓PI3K/Akt/mTOR signaling pathway, ↓Ras/Raf signaling pathway, ↑P53, ↑P21, ↑Bax, ↓Bcl‐2, ↑Caspase‐3/8/9 →Apoptosis	[101]
Ori + Venetoclax	*Rabdosia rubescens*	AML Cell Lines: THP‐1, OCI‐AML3, DNMT3A, NPM1, U‐937, MOLM13	↓AKT phosphorylation, ↑Bax, ↑Bim, ↓Mcl‐1 →Apoptosis	[102]
OP16 (A derivative of Oridonin)	*Rabdosia rubescens*	Human esophageal squamous cell carcinoma cell lines EC9706 and KYSE450	↓AKT phosphorylation, ↑Bax, ↓Bcl‐2, ↑Caspase‐3/9 →Apoptosis	[103]
Ori + Dox (Doxorubicin)	*Rabdosia rubescens*	Human osteosarcoma cell line Saos‐2	↑ROS, ↓Mcl‐1 →Apoptosis	[110]
Ori + Cetuximab	*Rabdosia rubescens*	Human laryngeal squamous cell carcinoma cell lines, HEp‐2 and Tu212	↓p‐JAK2, ↓p‐STAT3, ↓p‐PI3K, ↓p‐Akt, ↓MMP, ↑ROS →ER stress and apoptosis	[111]
TPL	*Tripterygium wilfordii*	Human leukemia chronic myelogenous leukemia K562 and acute promyelocytic leukemia HL‐60 cells	↓NRF2, ↑ROS, ↑Lipid oxidation, ↓GPX4 →Ferroptosis	[130]
BA	Birch, eucalyptus and plane trees	Human ovarian cancer cell line A2780	↑Caspase‐3/8/9, ↑Bax, ↓Bcl‐2 →Apoptosis	[145]
BA	Birch, eucalyptus and plane trees	Human gallbladder cancer cell lines NOZ, OCUG, SGC‐996, and EHGB‐1	↓SCD1, ↑Cyt‐C, ↑Bax, ↓Bcl‐2, ↑C‐PARP, ↑Caspase‐3/9 →Apoptosis	[146]
OA	Widely distributed in various medicinal plants	Hepatocellular carcinoma cell line HepG2	↓NF‐κB, ↓COX‐2, ↓PCNA, ↑Bax, ↓Bcl‐2, ↑Caspase‐3 →Apoptosis and autophagy	[165]
OA	Widely distributed in various medicinal plants	Hepatocellular carcinoma cell line SMMC‐7721	↑Akt/mTOR signaling pathway, ↓MMP, ↓Intracellular ATP ↑Bax, ↓Bcl‐2, ↑LC3‐II, ↑Beclin‐1 →Apoptosis and autophagy	[166]

Abbreviations: AIF, apoptosis inducing factor; AIM2, absent in melanoma 2; AKT, AKT serine/threonine kinase; Bad, BCL2 associated agonist of cell death; Bax, BCL2 associated X, apoptosis regulator; Bcl‐2, BCL2 apoptosis regulator; Bim, Bcl‐2 interacting mediator of cell death; CIRBP, cold inducible RNA binding protein; ClpP, caseinolytic mitochondrial matrix peptidase proteolytic subunit; COX‐2, cyclooxygenase‐2; C‐PARP, CLEAVED poly ADP‐ribose polymerase; Cyt‐C, cytochrome complex; DHA, dihydroartemisinin; DSB, double strand break; GPX4, glutathione peroxidase 4; JAK2, Janus kinase 2; LC3‐II, microtubule associated protein 1 light chain 3; Mcl‐1, MCL1 apoptosis regulator, BCL2 family member; MDA, malondialdehyde; MLKL, mixed lineage kinase domain like pseudokinase; MMP, mitochondrial membrane potential; mTOR, mechanistic target of rapamycin kinase; NDUFS3, NADH, ubiquinone oxidoreductase core subunit S3; NF‐κB, nuclear factor kappa b subunit 1; NRF2, NF‐E2‐related factor 2; P53, tumor protein P53; p70S6k, p70 ribosomal protein S6 kinase; p‐AKT, phosphorylation‐AKT serine/threonine kinase; p‐AMPK, phosphorylation‐adenosine 5′‐monophosphate (AMP)‐activated protein kinase; Parkin, parkin RBR E3 ubiquitin protein ligase; PARP‐1, poly ADP‐ribose polymerase‐1; PCNA, proliferating cell nuclear antigen; p‐ERK, phosphorylation‐extracellular‐regulated protein kinases; p‐ERK1/2 extracellular‐regulated kinase 1/2; p‐gp, P‐glycoprotein; PI3K, phosphatidylinositol‐4,5‐bisphosphate 3‐kinase catalytic subunit delta; PINK1, PTEN induced kinase 1; p‐JNK, phosphorylation‐mitogen‐activated protein kinase 8; PRIM2, DNA primase subunit 2; p‐STAT3, phosphorylation‐signal transducer and activator of transcription 3; Raf, Raf proto‐oncogene, serine/threonine kinase; Ras, Rat sarcoma; RIP‐1, receptor‐interacting protein‐1; RIPK, receptor‐interacting serine/threonine kinase; ROMO1, reactive oxygen species modulator 1; ROS, reactive oxygen species; SCD, Stearoyl‐CoA Desaturase; SDHB, succinate dehydrogenase complex iron sulfur subunit B; SLC7A11, solute carrier family 7 member 11; TFAM, transcription factor A, mitochondrial; UQCRFS1, ubiquinol‐cytochrome C reductase, rieske iron sulfur polypeptide 1; γH2AX, γH2A.X variant histone.

**TABLE 2:** In vivo studies of terpenoids as inducers of mitochondrial death in tumor cells Table.

Terpenoids	Plant sources	Cancer model	Mechanism and effects	Reference
DHA + DDP	*Artemisia annua*	BALB/c mice were subcutaneously injected with the pancreatic cancer cell line PANC1 cells	↓ATP, ↑ROS, ↓GPX4 →Ferroptosis	[67]
DHA + CDDP	*Artemisia annua*	The OSCC tumor‐xenograft mice	↑LC3‐II, ↑P62 →Mitophagy	[68]
DHA	*Artemisia annua*	Melanoma‐bearing BALB/c mice	↑IFN‐γ, ↓IL‐10, ↓p‐STAT3 →Apoptosis	[74]
DHA + DTX	*Artemisia annua*	Mice with metastatic breast cancer in situ derived from 4T1 cells	↑ROS, ↓MMP, ↑P53, ↑Cyt C release into the cytoplasm, ↑Caspase‐3 →Apoptosis	[75]
Ori	*Rabdosia rubescens*	Esophageal cancer xenograft in mice	↓PI3K/Akt/mTOR signaling pathway, ↓Ras/Raf signaling pathway, ↑P53, ↑P21, ↑Bax, ↓Bcl‐2, ↑Caspase‐3/8/9 →Apoptosis	[101]
Ori + Venetoclax	*Rabdosia rubescens*	AML mouse model	↓The growth of AML xenograft tumors in mice ↑The survival time of tumor‐bearing mice	[102]
TPL + JQ1 (A bromodomain and extra‐terminal domain inhibitor)	*Tripterygium wilfordii*	BALB/c mice were subcutaneously injected with the AML cell line MOLM‐13 cells	↑ROS, ↓c‐Myc, ↓Tumor weight and volume	[127]
CK21 (A derivative of Triptolide)	*Tripterygium wilfordii*	BALB/c mice were subcutaneously injected with the pancreatic cancer cell line PANC1 cells and AsPC‐1 cells	↓NF‐κB, ↑ROS ↓The growth of AML xenograft tumors in mice ↑The survival time of tumor‐bearing mice →Apoptosis	[128]
B5G1 (A betulinic acid analog)	Birch, eucalyptus, and plane trees	BALB/c mice were subcutaneously injected with the HepG2/ADM cells	↑PINK1, ↑p‐Parkin, ↓The growth of HepG2/ADM xenografts →Apoptosis and mitochondrial autophagy	[151]
OA	Widely distributed in various medicinal plants	DMBA‐induced hepatocellular carcinoma in situ model mice	↓NF‐κB, ↓Bcl‐2, ↑Caspase‐3, ↑Beclin‐1 →Apoptosis and autophagy	[165]

Abbreviations: ATP, adenosine 5′‐triphosphate; AKT, AKT serine/threonine kinase; Bax, BCL2 associated X, apoptosis regulator; Bcl‐2, BCL2 apoptosis regulator; Cyt‐C, cytochrome Complex; GPX4, glutathione peroxidase 4; LC3‐II, microtubule associated protein 1 light chain 3; MMP, mitochondrial membrane potential; mTOR, mechanistic target of rapamycin kinase; MYC, MYC proto‐oncogene; NF‐κB, nuclear factor kappa b subunit 1; P21, CKI; P53, tumor protein P53; P62, sequestosome 1; PI3K, phosphatidylinositol‐4,5‐bisphosphate 3‐kinase catalytic subunit delta; PINK1, PTEN induced kinase 1; p‐STAT3, phosphorylation‐signal transducer and activator of transcription 3; Raf, Raf proto‐oncogene, serine/threonine kinase; Ras, Rat sarcoma; ROS, reactive oxygen species.

## 6 Conclusion

Mitochondria are double‐membrane‐bound organelles that provide energy for cellular metabolism and play a key role in cell death. Growing evidence has indicated that mitochondrion‐dependent cell death plays a crucial role in tumors. Therefore, targeting mitochondria and inducing mitochondrial death in tumor cells is a promising therapeutic approach. Terpenoids, the most extensive category of natural compounds, have demonstrated the capability to induce mitochondrial apoptosis in malignant cells. However, the precise mechanism by which terpenoids cause mitochondrial cell death remains unclear. This review emphasizes the significance of selecting terpenoids in triggering mitochondrial death in cancer cells and their potential as therapeutic agents for cancer.

**FIGURE 2:**
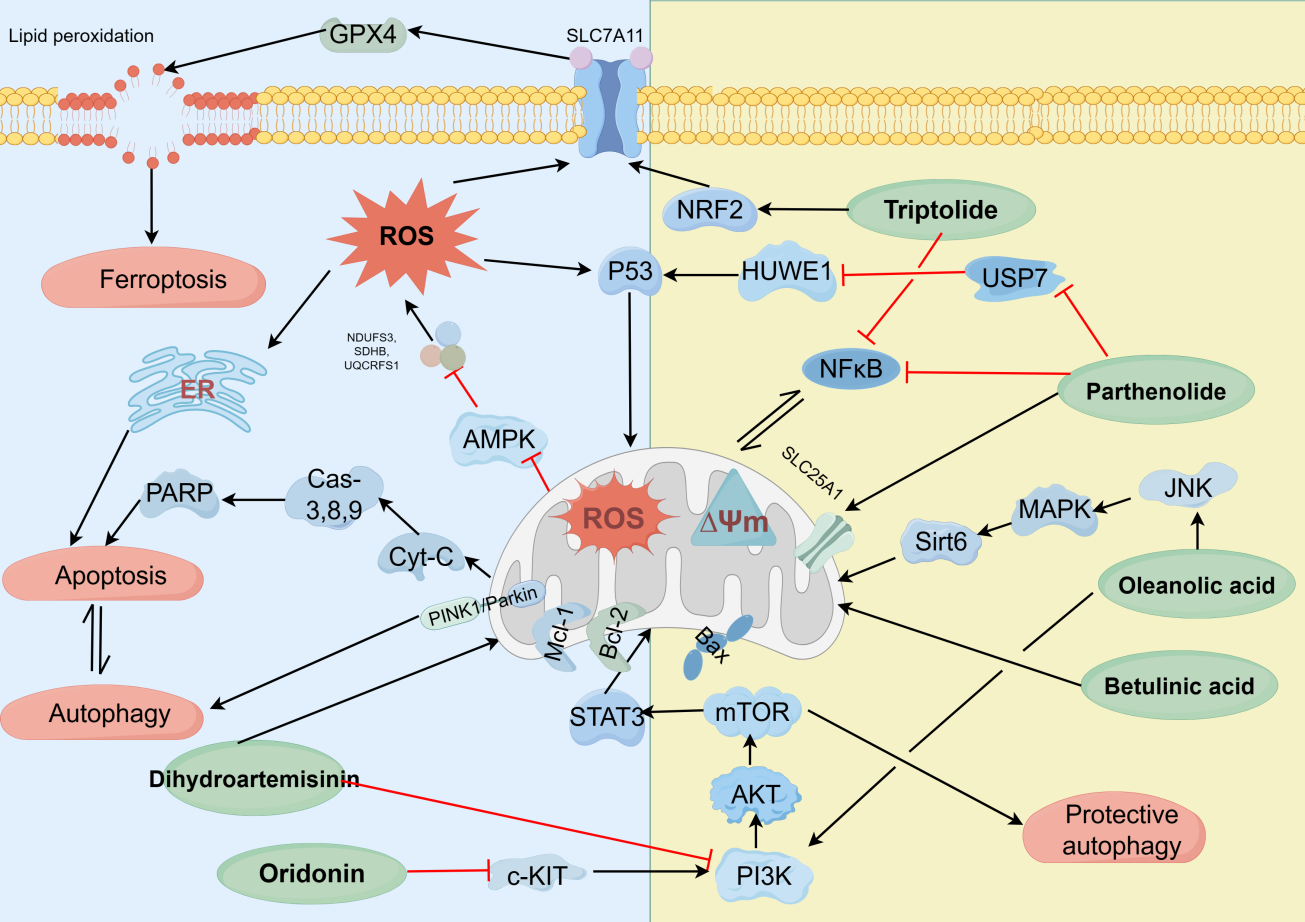
The effect of terpenoids on the mitochondrion‐dependent cell death signaling pathways. The terpenoids alone (green oval) exhibit the complex manner in which these secondary metabolites trigger the death of cells that rely on mitochondria via complicated signaling pathways like PI3K/AKT/mTOR, RIPK1‐RIPK3‐MLKL, MAPK/ERK/JNK, Ras/Raf, SIRT‐1/CAV‐1 axis, and NF‐κB. AKT, serine/threonine kinase; AMPK, AMP‐activated kinase; Bax, BCL2 associated X, apoptosis regulator; Bcl‐2, BCL2 apoptosis regulator; Cas‐3, Caspase‐3; Cas‐8, Caspase‐8; Cas‐9, Caspase‐9; c‐KIT, mast/stem cell growth factor receptor Kit; Cyt‐C, cytochrome complex; ER, sarcoplasmic/endoplasmic; GPX4, glutathione peroxidase 4; HUWE1, HECT, UBA, and WWE domain containing E3 ubiquitin protein ligase 1; JNK, c‐jun N‐terminal kinase; MAPK, mitogen‐activated protein kinase; Mcl‐1, MCL1 apoptosis regulator, BCL2 family member; mTOR, mechanistic target of rapamycin kinase; NF‐κB, nuclear factor kappa b subunit 1; NRF2, nuclear respiratory factors 2; P53, tumor protein P53; PARKIN, parkin RBR E3 ubiquitin protein; PARP, poly ADP‐ribose polymerase; PI3K, phosphatidylinositol‐4,5‐bisphosphate 3‐kinase catalytic subunit delta; PINK1, PTEN induced kinase 1; ROS, reactive oxygen species; SLC25A1, solute carrier family 25 member 1; SLC7A11, solute carrier family 7 member 11; Sirt6, Sirtuin 6; STAT3, signal transducer and activator of transcription 3; USP7, ubiquitin specific peptidase 7; ΔΨm, mitochondrial membrane potential.

The results of this review indicated that terpenoids trigger mitochondrial cell death in cancer cells through several mechanisms, including autophagy, ferroptosis, pyroptosis, necroptotic cell death, apoptosis, and necrosis mediated by mitochondrial permeability transition. It is intricately associated with multiple signaling pathways, including PI3K/AKT/mTOR, RIPK1‐RIPK3‐MLKL, MAPK/ERK/JNK, Ras/Raf, SIRT‐1/CAV‐1 axis, and NF‐κB (Figure 2). Interestingly, some studies have demonstrated that terpenoid‐induced mitochondrial apoptosis coincides with protective autophagy initiation facilitated by PINK1/Parkin or PI3K/Akt/mTOR. Consequently, elucidating the roles of signaling pathways involved in tumorigenesis may reveal new candidates for cancer treatment.

Another focus of the present review was the interplay between autophagy and apoptosis. Despite their differences in molecular biological pathways, they do affect each other in some cases. Autophagy has been shown to inhibit apoptosis, indicating that apoptosis becomes more probable when autophagy is suppressed. However, autophagy may facilitate apoptosis induction in certain instances. Deciphering the intricate association between autophagy and apoptosis provides new possibilities for cancer treatment. Protective autophagy suppression improves the cancer cells' sensitivity to apoptosis, whereas partial autophagy activation accelerates the onset of apoptosis.

Notably, structural modification, nanodelivery system adoption, and tandem administration of chemotherapeutic drugs improved the efficacy and bioavailability of terpenoids and mitigated chemoresistance. This reminds us of the feasibility of looking for anticancer drug leads among natural compounds.

Currently, research on the pharmacological actions of terpenoids and their derivatives primarily depends on in vivo and in vitro models for empirical data and theories regarding mechanisms, laying the foundation for clinical application. Unfortunately, the following issues remain unresolved: (a) terpenoid research remains in the preclinical phase, thereby lacking clinical trial data to support the studies; (b) the optimal dosage of terpenoids and their potential toxic side effects remain unclear; and (c) additional studies to elucidate the effects of terpenoids on mitochondrial cell death and reveal the complexity of the many signaling pathways involved are lacking. Several advanced techniques, such as bioinformatics, histology, and artificial intelligence, can be used to elucidate the effects of terpenoids on mitochondrial cell death and reveal the complexity of the signaling pathways involved before developing better anticancer treatments. In conclusion, terpenoids exhibit a great antitumor potential.

## Author Contributions

**Jianxin Guo:** writing – review and editing, writing – original draft, visualization. **Ming Huang:** writing – original draft, writing – review and editing. **Shuang Hou:** writing – review and editing, writing – original draft, data curation. **Jianfeng Yuan:** writing – review and editing, writing – original draft, data curation. **Xiaoyue Chang:** writing – review and editing. **Shuang Gao:** writing – review and editing. **Zhenhan Zhang:** writing – review and editing. **Zhongbing Wu:** funding acquisition, writing – review and editing, project administration, visualization. **Jing Li:** funding acquisition, writing – review and editing, project administration, conceptualization, formal analysis, supervision.

## Conflicts of Interest

The authors declare no conflicts of interest.

## Data Availability

Data sharing not applicable to this article as no datasets were generated or analysed during the current study.
